# Distinctive features gleaned from the comparative genomes analysis of clinical and non-clinical isolates of Klebsiella pneumoniae

**DOI:** 10.6026/97320630016256

**Published:** 2020-03-31

**Authors:** Jina Rajkumari, Supriyo Chakraborty, Piyush Pandey

**Affiliations:** 1 Department of Microbiology, Assam University, Silchar 788011, Assam, India; 2 Department of Biotechnology, Assam University, Silchar 788011, Assam, India

**Keywords:** Klebsiella pneumoniae, Comparative genomics, Codon usage bias

## Abstract

It is of interest to describe the distinctive features gleaned from the comparative genome analysis of clinical and non-clinical isolates of Klebsiella pneumoniae. The core genome of K. pneumoinae consisted of 3568 genes. Comparative genome analysis shows that mdtABCD, toxin-antitoxin systems are unique to clinical isolates and catB, benA, and transporter genes for citrate utilization are exclusive to non-clinical isolates. We further noted aromatic compound degrading genes in non-clinical isolates unlike in the later isolates. We grouped 88 core genes into 3 groups linked to infections, drug-resistance or xenobiotic metabolism using codon usage variation analysis. It is inferred using the neutrality plot analysis of GC12 with GC3 that codon usage variation is dominant over mutation pressure. Thus, we document data to distinguish clinical and non-clinical isolates of K. pneumoniae using comparative genomes analysis for understanding of genome diversity during speciation.

## Background

Klebsiella pneumoniae is a non-motile, gram-negative bacterium, which inhabits in diverse ecological niches ranging from soil to water and plants, and it is also opportunistic pathogen causing hospital-acquired disease in patients with the compromised immune system [1]. Human clinical isolates are considered as indistinguishable from environmental isolates with respect to their biochemical reactions and other attributes [2]. In plants, K. pneumoniae strains had been reported to fix nitrogen and promote growth of the plants [3]. Rajkumari et al. [4] had reported that Klebsiella strains have the ability to degrade hydrocarbons, including polyaromatic hydrocarbons (PAH). Previously, Holt et al. [5], have determined the diversity of genetic variation in specific genes associated with virulence and antibiotic resistance to track the emergence of invasive K. pneumoniae infections.

The advent of whole-genome sequencing along with reliable bioinformatics workflow has made possible to study the distinct genome patterns that may reflect the variation due to evolutionary pressures. Identification of conserved genomic core genes and pan genes from a collection of bacterial genomes depends on classifying orthologous genes based on similar sequences [6]. It provides valuable information and deeper insights into bacterial genome evolution, genes associated with host adaptation, virulence, and pathogenesis [7]. In previous work, the whole genome sequence analysis of clinical and environmental K. pneumoniae suggested that they are closely related but antibiotic resistance and virulence factors were more frequent in clinical isolates. In fact, the phylogenomics analysis of K. pneumoniae whole genome failed to result in any distinct segregation of clinical and non-clinical clades of K. pneumoniae, as the genomes originating from either group were mixed throughout the tree [8].

**Figure 1:**
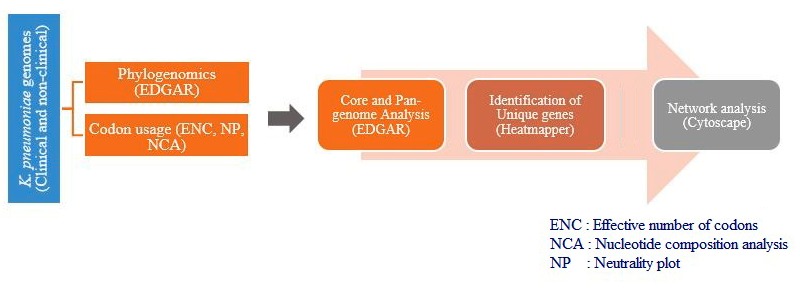
Flowchart of the workflow

Therefore, in this work, a genome analysis workflow was designed and used to identify the genes and their functions, which can be considered to resolve between clinical and non-clinical K. pneumoniae isolates (Figure 1). We used phylogenomics approach followed by the comparison of K. pneumoniae codon usage bias (CUB) pattern across the genes. In fact, the synonymous codons are known to be used non-randomly, and this unequal usage of the synonymous codon is called codon usage bias [9]. It is of interest to describe the distinctive features gleaned from the comparative genome analysis of clinical and non-clinical isolates of K. pneumoniae.

## Methodology:

### Genome comparison and Sequence Data 

Comparative genome analysis of a collection of K. pneumoniae (clinical and non-clinical genomes) was examined. A private project was created with standard K. pneumoniae genomes, comprising of non-clinical/environmental and clinical isolates. Phylogenetic relationships between thirty-eight K. Pneumoniae genomes were analyzed using EDGAR software platform (v.2.3) (http://edgar.computational.bio). EDGAR allows the calculation and identification of the core-pan genomes between different genomes. The nucleotide coding sequence (CDS) for eighty-eight genes, (24 drug resistant (DRGs), 16 infections related genes (IRGs) and 48 xenobiotic metabolism genes (XMGs) of five clinical and five non-clinical K. pneumoniae genomes having perfect start and stop codon were retrieved from IMG database (http://img.jgi.doe.gov) and gene details are given below.

### The effective number of codons

The observed effective number of codons (ENC) for each coding sequence of gene sets of K. pneumoniae was calculated using the formula given by Wright [10]. ENC value shows an inverse relationship with the degree of codon bias. ENC values range from 20 to 61, where low ENC value (<35) indicates high codon usage bias and high ENC value indicates low codon usage bias [10].

ENC = 2+9//F_2_+1//F_3_+5//F_4_+3//F_6_

where FK (K= 2, 3, 4, 6) is the mean of FK values for the K -fold degenerate amino acids.

### Nucleotide composition analysis

The overall nucleotide composition (A%, C%, T% and G%) and occurrence of overall frequency of the nucleotide (G+C) at first (GC1), second (GC2) and third (GC3) position of the synonymous codons were calculated in the coding sequences of the genes to quantify the extent of base compositional bias. The calculations were done using a Perl script developed by one of the authors (SC).

### Neutrality plot

The neutrality plot is a scattered plot, which is used to determine the role of directional mutational pressure against selection pressure during evolution. It is the regression of GC12 on GC3, as the synonymous mutation occurs in the 3^rd^ position of codon while non-synonymous mutations occur in the 1^st^ and 2^nd^ position. The non-synonymous mutation transforms the activity of the gene, which resulted from the alteration of amino acid sequence. In neutrality plot, if the regression line falls near the diagonal, it signifies weak external selection pressure and the role of mutation pressure is dominant.

### Software and Statistical Analysis

Heat map of the specific clinical and non-clinical genes was generated by Expression Heatmapper using an average linkage method with Euclidean distance [11]. A network of genes was created for selected unique genes of clinical and non-clinical isolates by Cytoscape 3.4.0 with GeneMANIA plugin. The node degree distribution of the complex protein-protein interaction network was obtained from Cytoscape by Network analyzer [12]. A PERL program was developed to estimate the genetic codon usage bias indices and the selection pressure on the coding sequence of K. pneumoniae genes. Correlation analysis was performed to identify the degree of relationship between two parameters by Karl Pearson's method. The significance of the correlation coefficient was tested by t-test for (n-2) degrees of freedom at p<0.01 or p<0.05. Statistical analyses were performed using IBM SPSS version 21.0 for windows.

## Results 

**Figure 2:**
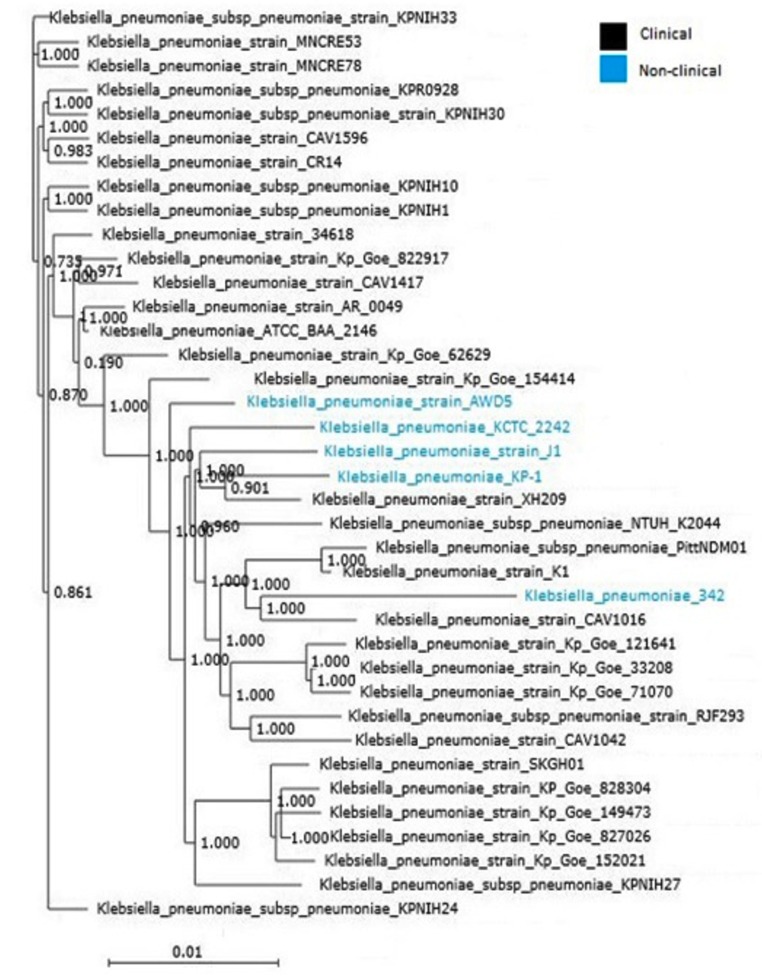
Phylogenetic tree constructed from the core genome of K. pneumoniae genomes. The scale bar, 0.01 corresponds to the substitution per amino acid within the coding regions of the core genome.

Phylogenomic relationship of K. pneumoniae (clinical and non-clinical) genomes was examined from the deduced amino acid sequences of the core genomes and resolved the close relationship among non-clinical and clinical isolates. The main features of the genome sequences of K. pneumoniae non-clinical strain and clinical strain are summarized below (Table 1). The tree was built out of core genome taking 3568 genes per genome, 135584 in total. There was no clear separation of clades between clinical and non-clinical isolates (Figure 2). Phylogenomic analysis of the core genomes 48 clinical and 29 environmental K. pneumoniae isolates had demonstrated that the isolates were intermixed and failed to result in any distinct segregation of clinical and non-clinical clades of K. pneumoniae [8].

**Figure 3:**
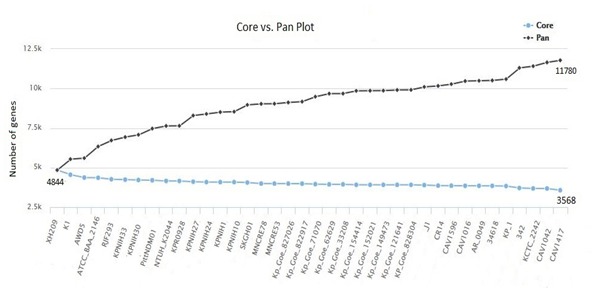
Core vs pan-genome development plot of K. pneumoniae genomes (EDGAR 2.2 software platform)

Based on the phylogenomics results, genomes (clinical and non-clinical) were selected for further analysis and orthologous genes in K. pneumoniae were calculated. Core genome analyses of the K. pneumoniae genomes showed that it consisted of 3568 conserved genes. While the pan-genome appeared to grow rapidly and the core genome was limited to less than 4000 genes. There are several Pan-genome analysis pipelines are available [13,14,15, 16] however we have used EDGAR pipeline for studying genetic variation, and function enrichment analyses of the gene clusters. Core versus pan-genome development analysis of K. pneumoniae genomes revealed that 3568 formed the core genome while 11780 genes formed the pan-genome when K. pneumoniae AWD5 is used as the reference genome (Figure 3). The genes in AWD5 strain cover 89.45% of the coding genes in the genome. Average nucleic acid identities (ANI) of AWD5 with K. pneumoniae KP-1, K. pneumoniae ATCC-BAA 2146 and K. pneumoniae subsp. pneumoniae NTUH-K2044 revealed 99% sequence homologies and 94.02% with K. pneumoniae 342. The genomes of AWD5 and ATCC BAA-2146 (clinical strain) appear to be most similar by sharing 4529 orthologs whereas 4442 orthologs were found between AWD5 and the environment isolate KP-1. Pan-genome of six K. pneumoniae strains was reported to be consisted of 4,829 core genes, such a high percentage signifies a high rate of conservation among the strains. Previously, some studies had reported that phenotypic and genetic features of K. pneumoniae of environmental and clinical origin were similar and therefore, the isolates cannot be distinguished [2, 17].

## Gene content analysis of K. pneumoniae

By investigating the presence and absence of genes in K. pneumoniae, most of the regions within the genomes were found to be conserved, including the virulence genes present in the clinical strains regardless of disease source. The orthologs include components of regulatory pathways such as basic transcriptional machinery, DNA relication, homologous recombination, mismatch repair, nucleotide excision repair, bacterial secretion system and protein export. Genes attributable to the production of indole-3-acetic acid (IAA) (ipdC), solubilization of phosphate (pqqABCDEF, phn and pho gene clusters, pstBACS), synthesis of siderophore (ent, fep gene clusters), acetoin and 2,3-butanediol (alsDSR, budC) are found to be conserved. It also revealed the presence of multicomponent nitrate or nitrite transport system. More than 13 genes involved in benzoate (ben genes), catechol (cat genes), protocatechuate (pca genes) were conserved in the core genome of K. pneumoniae.

**Figure 4:**
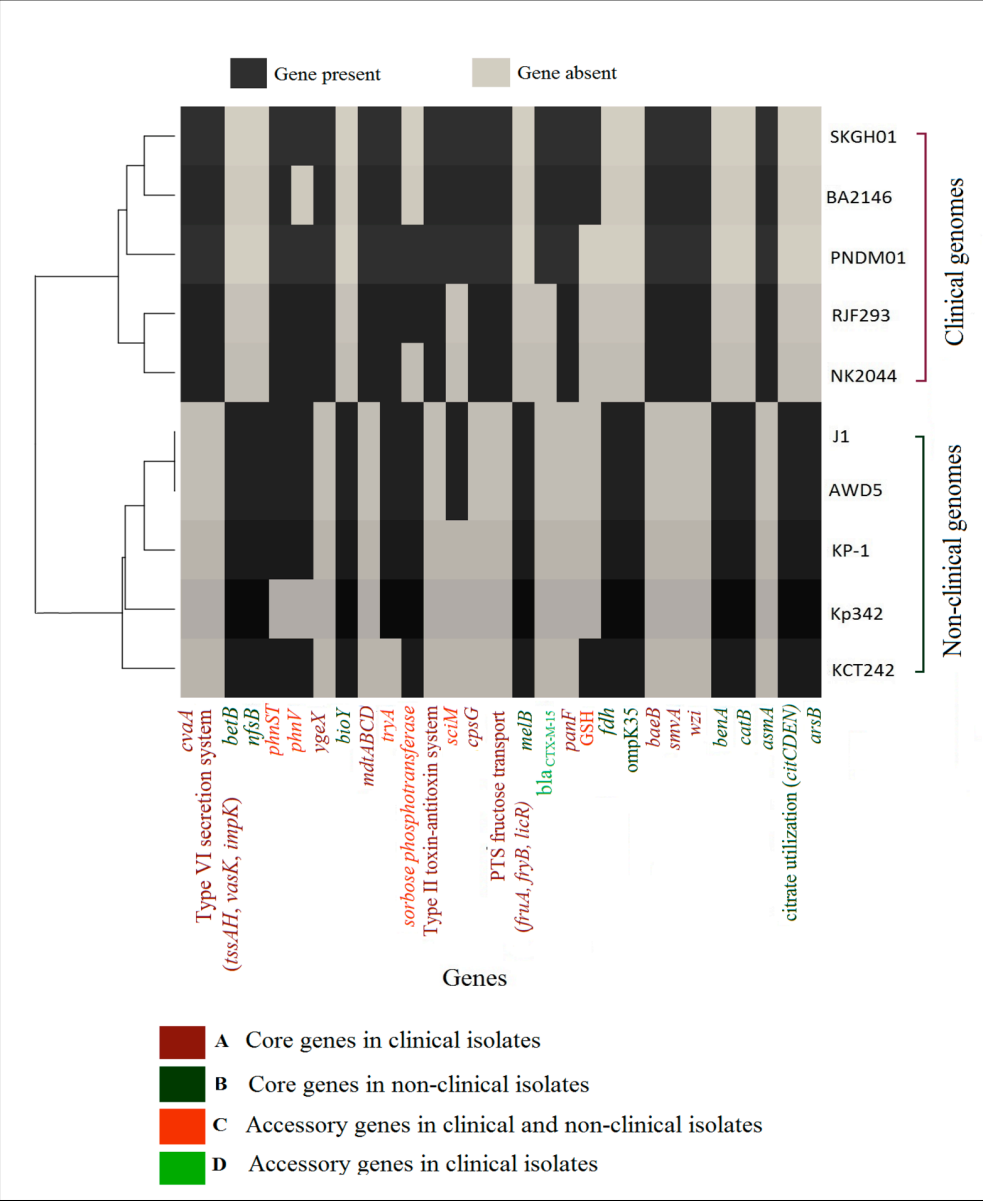
Dendogram showing the relationship between genomes based on the presence and absence of genes designated as group specific.

**Figure 5:**
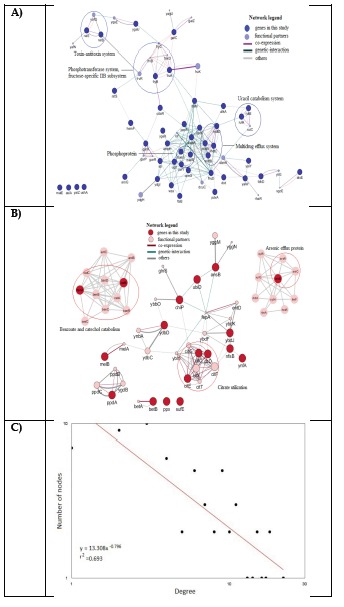
Cytoscape and GeneMANIA interaction network of known (a) clinical and (b) non-clinical unique core genes with co-expression and genetic interaction of other related genes. Nodes represent proteins and edges represent interactions between the nodes. Power law node-degree distribution of the networks in genes of clinical and non-clinical genomes (c and d). The node degree (k) is represented on the x-axis and number of nodes with a particular k is represented on the y-axis. The Pearson correlation coefficient values (r2) and the probability of degree distributions P(k) are shown. The red lines indicate the power law. The R-squared is computed on log values.

Further, to obtain the unique genes, the genomes were segregated into two groups i.e., clinical and non-clinical, taking five genomes for each group and analyzed separately. In clinical isolates, such core genes included virulence factor capsule assembly protein, multidrug transporter subunit (mdtABCD), type II toxin-antitoxin systems, and type VI secretion system (TVISS). TVISS were found to be shared among clinical and non-clinical isolates however, at least eight CDSs determined in clinical genomes involved in TVISS, were not found in non-clinical isolates. It has been reported that an environmental isolate Kp342 and clinical isolate MGH78578 seemed to share core components of TVISS [18]. The unique core genome of non-clinical isolate consisted of aromatic compound degrading genes catB, benA and several transporters including genes for citrate utilization. This was in agreement to the hypothesis that environmental isolates are more versatile for their catabolic processes, as the ability to degrade organic compounds govern the evolution of novel catabolic abilities of bacteria that can survive in different habitats [19]. We took the presence/absence of such individual genes as the categorical analyst parameter subjected to build a phylogenetic tree. And showed that the strains from similar clinical and environmental sources were not linked than those from different sources (Figure 4). The core genes discriminated the clinical and environmental genomes and accessory genes segregated the strains within the group. Based on the gene functions, several gene sets were experimented which may resolve clinical and non-clinical isolates. Accordingly, one set of unique core genes in non-clinical isolates and clinical isolates is given in the figure 4 on the basis of which genomes were resolved into two distinct categories, further few genes were useful for resolving the genomes within the particular group. Virulence factor (galF, wcaI, wcaG, wzb, wzc, manBC), allantoin utilization genes (gcl, allABCDS, ybbW), TVISS (impL, vgrG, tssFGJ) and other antibiotic resistance genes were also observed as accessory genes in ten K. pneumoniae. The clinical genomes possessed multidrug resistance gene blaCTX-M-15 as accessory gene; this is an indication of horizontal gene transfer that might have occurred between virulent and multidrug-resistant K. pneumoniae strains [20]. This finding agrees with the previous study, which reported that resistance to multiple antibiotics was found to be more frequent in clinical origin than that of environmental origin [21]. The gene interaction network of specific core genes of clinical and non-clinical K. pneumoniae genomes was analyzed in cytoscape and GeneMANIA (Figure 5) and data is given in supplementary file. It was noted that the functional partners were not part of the core genome of the respective group; however, it surely had interactions, and hence influence the functions of these genes.

## Extent of Codon usage variation in K. pneumoniae

**Figure 6:**
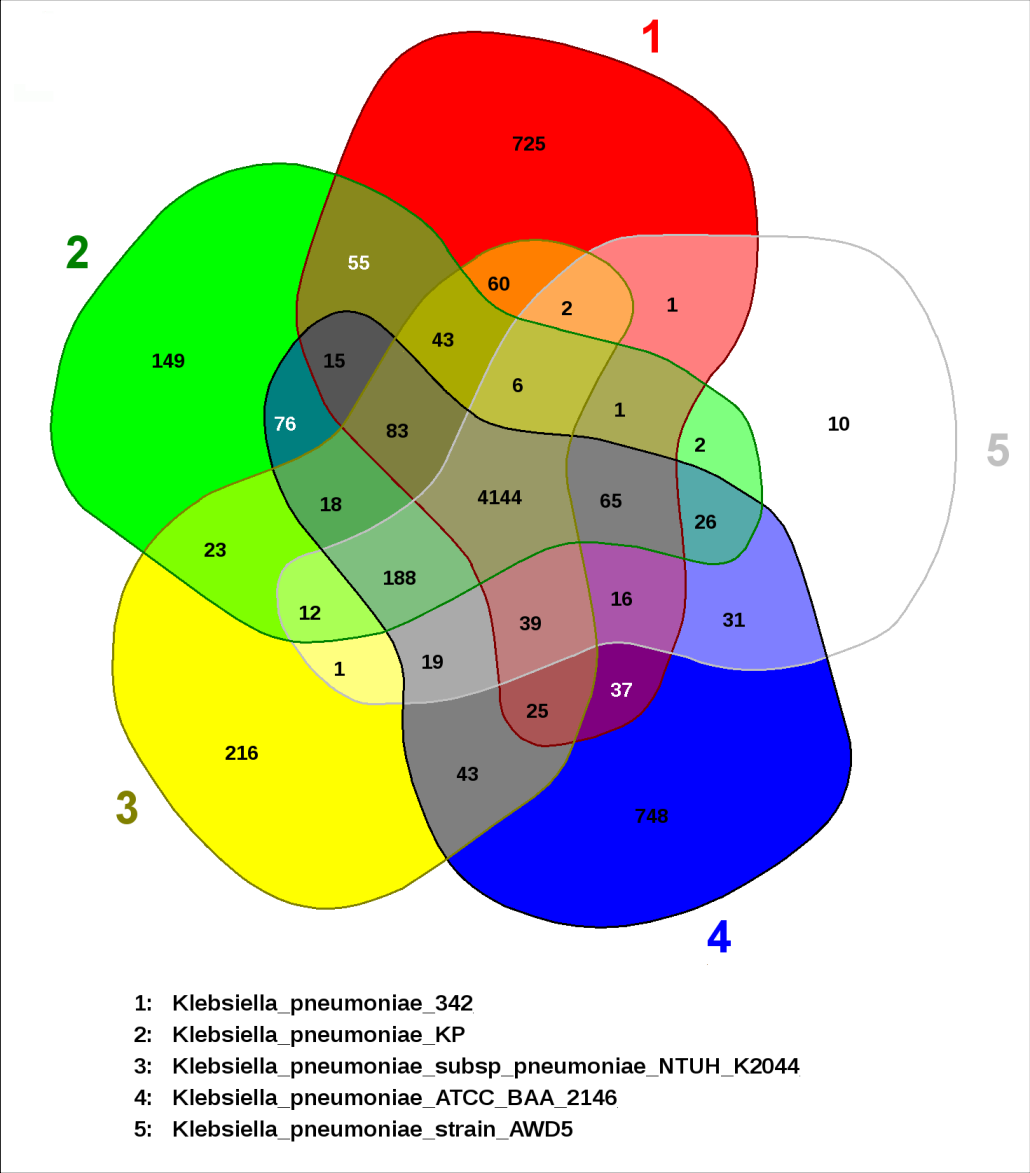
Visualizing common gene pools in five K. pneumoniae strains by Venn diagrams (EDGAR 2.2 software platform).

In order to analyze codon usage variation five K. pneumoniae genomes were selected considering their level of similarity. The data set was restricted to 5 genomes to avoid non-ambiguity. According to the EDGAR interface, genes were placed at intersections of the Venn diagram only if they were reciprocal matches. The analysis utilizes all CDS of the genomes and it is not restricted to the core genome. Among the K. pneumoniae strains, AWD5 chromosome shared 31 orthologous CDS with BA2146 and further 188 CDS conjointly with the strain KP-1. Moreover, 65 orthologous CDS are found to be shared conjointly by environmental isolates AWD5, KP-1 and Kp342 (Figure 6). Also, it indicated that AWD5 has 10 singleton genes. Besides, the singletons that resemble to the genes without reciprocal best hit to another genome, as orthologs does not necessarily have to be a proper singleton.

The codon usage variation was studied for eighty-eight genes (24 DRGs, 16 IRGs, and 48 XMGs) from five K. pneumoniae genomes of non-clinical origin (Kp342, KP-1, AWD5) and clinical origin (NK2044, BA2146) core genome. Selected genes details are given in the supplementary file (Table 2).

To quantify the extent of variation in codon usage among different genomes of K. pneumoniae, the effective number of codon (ENC) values for three gene sets of each genome was calculated. The ENC is a non-directional measure of codon usage bias, widely used to measure for individual genes. The ENC values among five K. pneumoniae strains ranged from 37.1 to 39.71, indicating low codon usage bias (Table 3) for IRGs, DRGs and XMGs respectively. (ENC < 40) represents stable ENC values and indicates that there is almost no variation of codon usage bias among the genes of K. pneumoniae. It also indicates conserved genomic composition among different K. pneumoniae genomes. Correlation analysis between codon usage bias and compositional properties of GC content were analyzed to understand the effect of base composition on codon usage bias. Negative correlation was observed between ENC and GC composition (Table 4). These results suggested that natural selection might have played an important role in codon usage pattern across the genes. Our results show that codon usage bias and gene expression among different K. pneumoniae genomes was lower, slightly biased in the genes [22].

Nucleotide composition analyses of coding sequences of IRGs, DRGs, and XMGs (Table 3) showed mean percentage of GC and AT compositions was 59.44% and 40.5% in IRGs, 59.36% and 40.66% in DRGs and 61.66% and 38.34% in XMGs respectively. Genes were GC-rich, GC content at the third position was higher than at the first and second codon position and the greatest difference of GC content was found between the second and the third codon positions. Hence, the overall nucleotide composition suggested that the nucleotide C and G occurred more frequently compared to A and T in the coding sequences and it is expected that G/C-ended codons might be preferred over A/T ended codons in the genomes. The difference in frequencies of A and T and to that of G and C were not the same which indicates that natural selection might have played a role in codon usage pattern [22].

**Figure 7:**
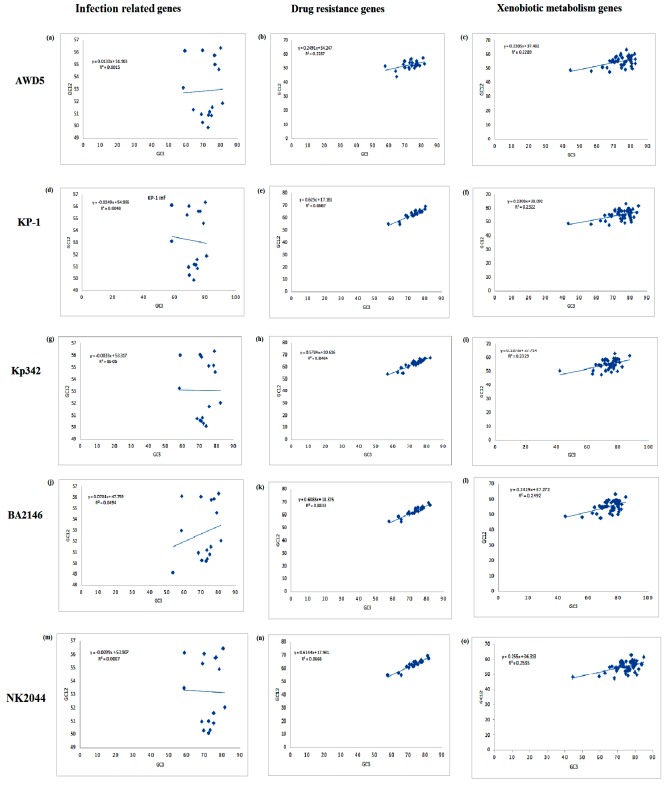
Neutrality plot of infection-related genes, drug resistance gene and xenobiotic metabolism genes of K. pneumoniae genome AWD5 (a-c), KP-1 (d-f), Kp342 (g-i), BA2146 (j-l), NK2044 (m-o). Individual genes are plotted based on the mean GC content in the first and second codon position (GC12) versus GC content of the third codon position (GC3).

A neutrality plot analysis of GC12 (average value of GC1 and GC2) versus GC3 (Figure 7) was drawn to characterize the correlation of the three codon position of GC 23], and to estimate the influence of selection and mutation pressure on codon usage bias of IRGs, DRGs and XMGs of five K. pneumoniae strains [24]. The regression coefficient of GC12 on GC3 for IRGs of the K. pneumoniae strains AWD5, BA2146, KP-1, Kp342 and NK2044 were 0.0133, 0.0701, 0.0249, 0.0033 and 0.0099 indicating relative neutrality of 1.33%, 7.01%, 2.49%, 0.33%, and 0.99% respectively. The GC12 was influenced by mutation pressure and natural selection with a ratio of 1.33/98.67=0.014, 7.01/92.99=0.75, 2.49/97.51=0.026, 0.33/99.67=0.003, and 0.99/99.01=0.009 for AWD5, BA2146, KP-1, Kp342 and NK2044 respectively. The subsequent correlation analysis revealed positive correlation between GC12 and GC3 in DRGs and XMGs (Table 5). These results suggest that natural selection played a major role while mutation pressure played minor role in shaping the composition of coding sequence of K. pneumoniae [25].

## Conclusions:

A comparative genome analysis shows that clinical and non-clinical strains of K. pneumonia are similar and are not separated by phylogeny. However, gene level comparison help distinguish these isolates where mdtABCD, toxin-antitoxin systems are distinctive to clinical isolates and catB, benA, transporter genes for citrate utilization are limited to non-clinical isolates. Thus, these data help distinguish clinical and non-clinical isolates of K. pneumoniae towards the understanding of genome diversity during speciation.

## Figures and Tables

**Table 1:** Genomic features and comparison of K. pneumoniae genomes used for analysis

Strain	Size (bp)	CDS	GC (%)	Source	References
K. pneumoniae AWD5 (AWD5)	4807409	4636	58.18%	Non-clinical	Rajkumari et al. 2017
K. pneumoniae 342 (Kp342)	5641239	5768	56.87%	Non-clinical	Fouts et al. 2008
K. pneumoniae SKGH01 (SKGH01)	6088457	5777	56.54%	Clinical	Alfaresi 2018
K. pneumoniae subsp. pneumonia PittNDM01 (PNDM01)	5812304	5529	56.78%	Clinical	Doi et al. 2014
K. pneumoniae J1 (J1)	5406866	5039	57.24	Non-clinical	Pang et al. 2016
K. pneumoniae strain KCTC-2242 (KCT242)	5462423	5152	57.28	Non-clinical	Shin et al. 2012
K. pneumoniae subsp. pneumonia RJF293 (RJF293)	5450593	5077	57.2	Clinical	Wang et al. 2018
K. pneumoniae strain KP-1(KP-1)	5131085	4755	57.6	Non-clinical	Lee et al. 2013
K. pneumoniae NTUH-K2044 (NK2044)	5248520	5,006	57.7	Clinical	Wu et al. 2009
K. pneumoniae ATCC BAA- 2146 (BA2146)	5680367	5552	56.9	Clinical	Hudson et al. 2014
K. pneumoniae subsp. pneumoniae KPNIH10	5395263	5653	57.14	clinical	NZ_CP007727
K. pneumoniae subsp. pneumoniae KPNIH1	5394056	5654	57.14	clinical	NZ_CP008827
K. pneumoniae subsp. pneumoniae strain KPNIH33	5574202	5500	57.21	clinical	NZ_CP009771
K. pneumoniae subsp. pneumoniae KPNIH27	5241638	5932	56.71	clinical	NZ_CP007731
K. pneumoniae subsp. pneumoniae KPNIH24	536164	5590	57.12	clinical	NZ_CP008797
K. pneumoniae subsp. pneumoniae KPNIH30	5306618	5346	57.26	clinical	NZ_CP009872
K. pneumoniae strain XH209	5118878	4881	57.63	clinical	NZ_CP009461
K. pneumoniae strain CAV1016	5387681	5391	57.22	clinical	NZ_CP017934
K. pneumoniae strain CAV1042	5424949	5500	56.81	clinical	NZ_CP018671
K. pneumoniae strain CAV1596	5402147	5450	57.14	clinical	NZ_CP011647
K. pneumoniae strain CAV1417	5208900	5,373	56.98	clinical	NZ_CP018352
K. pneumoniae Kp_Goe_71070	5497083	5434	56.95	clinical	NZ_CP018450
K. pneumoniae Kp_Goe_827026	5373056	5662	56.62	clinical	NZ_CP018707
K. pneumoniae Kp_Goe_149473	5373056	5677	56.62	clinical	NZ_CP018686
K. pneumoniae Kp_Goe_152021	5373055	5673	56.62	clinical	NZ_CP018713
K. pneumoniae Kp_Goe_828304	5373056	5683	56.62	clinical	NZ_CP018719
K. pneumoniae MNCRE78	5454003	5584	56.86	clinical	NZ_CP018428
K. pneumoniae MNCRE53	5490693	5627	56.87	clinical	NZ_CP018437
K. pneumoniae Kp_Goe_154414	5159815	5618	56.62	clinical	NZ_CP018337
K. pneumoniae Kp_Goe_62629	5423372	5607	57.16	clinical	NZ_CP018364
K. pneumoniae Kp_Goe_33208	5497872	5429	56.96	clinical	NZ_CP018447
K. pneumoniae Kp_Goe_822917	5294741	5360	57.21	clinical	NZ_CP018438
K. pneumoniae strain CR14	5470889	5869	56.78	clinical	NZ_CP015392
K. pneumoniae Kp_Goe_121641	5478335	5390	56.96	clinical	NZ_CP018735
K. pneumoniae subsp. pneumoniae KPR0928	5309305	5286	57.32	clinical	NZ_CP008831
K. pneumoniae strain 34618	5313576	5487	57.21	clinical	NZ_CP010392
K. pneumoniae strain AR0049	5435743	5661	56.98	clinical	NZ_CP018816
K. pneumoniae strain K1	5453585	5237	57.44	clinical	NZ_LOEJ01000001

**Table 2:** Selected gene of K. pneumoniae and functions

Sl.no	Gene	Protein function
1	lepA	GTP-binding protein
2	ureC	urease subunit alpha
3	groL	chaperonin GroEL
4	fimA	type 1 major fimbrial subunit precursor
5	fimD	putative export and assembly usher protein of type 1 fimbriae
6	fimG	type 1 fimbrial minor component
7	fimH	type 1 fimbrial adhesin precursor
8	cyaA	adenylate cyclase
9	sdhA	succinate dehydrogenase subunit A
10	tolC	outer membrane channel protein
11	norW	nitric oxide reductase
12	norV	anaerobic nitric oxide reductase flavorubredoxin
13	RpoS	RNA polymerase, sigma 38 subunit
14	RpoN/SigL	RNA polymerase, sigma 54 subunit
15	yegQ	putative protease
16	LuxS	S-ribosylhomocysteine lyase/quorum-sensing autoinducer 2 (AI-2) synthesis protein
17	arnA	UDP-4-amino-4-deoxy-L-arabinose formyltransferase
18	arnB	UDP-4-amino-4-deoxy-L-arabinose-oxoglutarate aminotransferase
19	arnC	undecaprenyl-phosphate 4-deoxy-4-formamido-L-arabinose transferase
20	arnF	undecaprenyl phosphate-alpha-L-ara4N flippase subunit
21	arnT	4-amino-4-deoxy-L-arabinose transferase
22	amiA	N-acetylmuramoyl-L-alanine amidase
23	amiB	N-acetylmuramoyl-L-alanine amidase
24	sapB	cationic peptide transport system permease protein
25	sapC	cationic peptide transport system permease protein
26	sapD	cationic peptide transport system ATP-binding protein
27	sapF	cationic peptide transport system ATP-binding protein
28	basS	two-component system, OmpR family, sensor histidine kinase
29	mraY	Phospho-N-acetylmuramoyl-pentapeptide-transferase
30	oppA	oligopeptide transport system substrate-binding protein
31	oppB	oligopeptide transport system permease protein
32	oppC	oligopeptide transport system permease protein
33	oppD	oligopeptide transport system ATP-binding protein
34	oppF	oligopeptide transport system ATP-binding protein
35	ppiA	peptidyl-prolyl cis-trans isomerase A (cyclophilin A)
36	phoP	two-component system, OmpR family, response regulator
37	phoQ	two-component system, OmpR family, sensor histidine kinase
38	ftsI	peptidoglycan synthetase
39	dnaK	molecular chaperone
40	marA	transcriptional regulator, AraC family
41	benA/xylX	benzoate/toluate 1,2-dioxygenase alpha subunit,
42	catA	catechol 1,2-dioxygenase
43	catB	muconate cycloisomerase
44	catC	muconolactone D-isomerase
45	pcaB	3-carboxy-cis,cis-muconate cycloisomerase,
46	pcaD	3-oxoadipate enol-lactonase
47	pcaG	protocatechuate 3,4-dioxygenase alpha subunit,
48	pcaH	protocatechuate 3,4-dioxygenase beta subunit,
49	pcaI	3-oxoadipate CoA-transferase alpha subunit,
50	pcaJ	3-oxoadipate CoA-transferase beta subunit,
51	paaA	ring-1,2-phenylacetyl-CoA epoxidase subunit,
52	paaF	enoyl-CoA hydratase
53	paaH	3-hydroxybutyryl-CoA dehydrogenase,
54	paaK	phenylacetate-CoA ligase
55	paaZ	oxepin-CoA hydrolase / 3-oxo-5,6-dehydrosuberyl-CoA semialdehyde dehydrogenase,
56	gabD	succinate semialdehyde dehydrogenase,
57	gabT	4-aminobutyrate aminotransferase / (S)-3-amino-2-methylpropionate transaminase,
58	glnB	nitrogen regulatory protein P-II family,
59	Gst	glutathione S-transferase
60	hpaA	AraC family transcriptional regulator, 4-hydroxyphenylacetate 3-monooxygenase operon regulatory protein,
61	hpaC	4-hydroxyphenylacetate 3-monooxygenase reductase component,
62	hpaD/hpcB	3,4-dihydroxyphenylacetate 2,3-dioxygenase,
63	hpaF	5-carboxymethyl-2-hydroxymuconate delta isomerase,
64	hpaH	2-oxohept-3-enedioate hydratase,
65	hpaG	5-carboxy-2-oxohept-3-enedioate decarboxylase HpaG1 subunit
66	hpaG	5-carboxy-2-oxohept-3-enedioate decarboxylase HpaG2 subunit
67	mhpA	3-hydroxyphenylpropionate hydroxylase,
68	mhpB	2,3-dihydroxyphenylpropionate 1,2-dioxygenase,
69	mhpC	2-hydroxy-6-ketonona-2,4-dienedioate hydrolase,
70	mhpD	2-keto-4-pentenoate hydratase
71	mhpE	4-hydroxy 2-oxovalerate aldolase
72	mhpF	acetaldehyde dehydrogenase
73	entA	2,3-dihydro-2,3-dihydroxybenzoate dehydrogenase,
74	entB	bifunctional isochorismate lyase / aryl carrier protein,
75	entC	isochorismate synthase
76	entD	enterobactin synthetase component D,
77	fepA	outer membrane receptor for ferrienterochelin and colicins,
78	fepB	iron complex transport system substrate-binding protein,
79	fepC	iron complex transport system ATP-binding protein,
80	fepD	iron complex transport system permease protein,
81	pqqB	pyrroloquinoline quinone biosynthesis protein B,
82	pqqC	pyrroloquinoline-quinone synthase,
83	pqqD	pyrroloquinoline quinone biosynthesis protein D,
84	pqqE	pyrroloquinoline quinone biosynthesis protein E,
85	pstA	phosphate ABC transporter membrane protein 2, PhoT family,
86	pstB	phosphate ABC transporter ATP-binding protein, PhoT family,
87	pstC	phosphate ABC transporter membrane protein 1, PhoT family,
88	pstS	phosphate ABC transporter substrate-binding protein, PhoT family,

**Table 3:** Nucleotide composition analysis in the coding sequence of gene sets in K. pneumoniae strains

Sl. No.	A	T	G	C	AT%	GC%	GC1%	GC2%	GC3%	AT3%	GC12%	ENC
Infection-related gene												
AWD5	313.1	259.9	403.8	435.6	40.7	59.3	63.4	42.3	72.1	27.9	52.8	38.19
KP-1	308.8	260.9	406.6	431.2	40.5	59.5	63.8	42.5	72.1	27.8	52.8	38.46
Kp342	299.7	247.1	385.8	415.4	40.6	59.5	63.9	42.2	72.2	27.8	53.1	37.56
BA2146	296.3	248.1	386.2	411.3	41	59	63.8	41.7	71.4	28.6	52.9	39.21
NK2044	297.2	248.8	390.1	415.4	40.5	59.5	63.8	42.5	72.2	27.7	53.2	38.64
Mean	303.02	252.96	394.5	421.78	40.66	59.36	63.74	42.24	72	27.96	52.96	38.91
SD	7.501	6.827	9.96	10.85	0.207	0.219	0.195	0.328	0.339	0.365	0.182	0.61
Drug resistance gene												
AWD5	214.8	210.8	302.3	323.2	40.4	59.5	64.4	40.6	73.5	26.4	52.6	39.23
KP-1	213.4	211	302.5	321.2	40.5	59.5	64.5	40.7	73.3	26.6	62.9	39.17
Kp342	220.5	218.2	310	325.5	40.7	59.3	64.3	40.9	72.6	27.3	62.6	39.71
BA2146	214.9	212.9	301.6	319.7	40.6	59.3	64.3	40.8	72.9	27.1	62.7	39.5
NK2044	213.9	211	303.4	322.1	40.3	59.6	64.6	40.7	73.5	26.5	63.1	39.3
Mean	215.5	212.78	303.96	322.34	40.5	59.44	64.42	40.74	73.16	26.78	60.78	39.38
	Xenobiotic metabolism gene											
SD	2.864	3.148	3.437	2.182	0.158	0.134	0.131	0.114	0.397	0.396	4.577	0.22
												
AWD5	183.5	168.7	285.3	296.6	38.3	61.7	67.1	43.4	74.6	25.3	55.3	38.93
KP-1	178.2	161.3	271.7	285.7	38.3	61.7	67.1	43.5	74.5	25.4	55.3	39.1
Kp342	179.1	162.8	270.6	284.2	38.5	61.5	66.9	43.5	73.8	26.1	55.2	39.36
BA2146	178.3	160.8	271.6	285.3	38.3	61.7	67.1	43.5	74.5	25.5	55.2	37.1
NK2044	177.8	160.6	271.3	284.8	38.3	61.7	67.1	43.4	74.4	25.6	55.3	38.19
Mean	179.38	162.84	274.1	287.32	38.34	61.66	67.06	43.46	74.36	25.58	55.26	38.52
SD	2.351	3.387	6.276	5.218	0.089	0.089	0.089	0.055	0.321	0.311	0.054	0.91
SD: standard deviation, GC12: the average of GC contents at first and second codon positions.

**Table 4:** Summary of correlation analysis between ENC and various GC content

ENC	GC	GC1	GC2	GC3
AWD5 r	-0.414*	-0.443*	-0.184	-0.245
p value	0.044	0.03	0.388	
				0.249
KP-1 r	-0.440*	-0.440*	-0.212	-0.265
p value	0.031	0.032	0.319	0.211
KP342 r	-0.386	-0.511**	0.148	-0.298
p value	0.057	0.009	0.481	0.148
BA2146 r	-0.466*	-0.474*	-0.079	-0.356
p value	0.022	0.019	0.715	0.087
NK2044 r	-0.441*	-0.450*	-0.228	-0.265
p value	0.031	0.027	0.284	0.211
				
ENC	GC	GC1	GC2	GC3
AWD5 r	-0.275	-0.426	0.206	-0.155
p value	0.302	0.1	0.444	0.566
KP-1 r	0.047	-0.312	0.311	0.017
p value	0.863	0.239	0.24	0.949
KP342 r	0.042	-0.208	0.014	0.184
p value	0.877	0.439	0.958	0.496
BA2146 r	-0.527*	-0.520*	0.127	-0.437
p value	0.036	0.039	0.639	0.091
NK2044 r	-0.028	-0.359	0.32	-0.063
p value	0.918	0.171	0.227	0.817
				
ENC	GC	GC1	GC2	GC3
AWD5 r	-0.348*	0.026	-0.22	-0.503**
p value	0.015	0.86	0.133	0
KP-1 r	-0.369**	-0.01	-0.194	-0.516**
p value	0.01	0.947	0.186	0
KP342 r	-0.384**	0.056	-0.203	-0.580**
p value	0.007	0.708	0.167	0
BA2146 r	-0.395**	-0.018	-0.272	-0.520**
p value	0.005	0.902	0.061	0
NK2044 r	-0.339*	0.03	-0.243	-0.475**
p value	0.019	0.839	0.096	0.001
*P < 0.05, **P < 0.001

**Table 5:** Summary of correlation analysis of GC12 Vs GC3

	GC12	AWD5 GC3	KP-1 GC3	Kp342 GC3	BA2146 GC3	NK2044 GC3
Drug resistance gene	r	0.478*	0.454*	0.305	0.440*	0.429*
	p	0.018	0.026	0.138	0.031	0.037
Infection related gene	r	0.038	-0.069	-0.009	0.222	-0.026
	p	0.888	0.8	0.974	0.408	0.923
Xenobiotic metabolism gene	r	0.478**	0.482**	0.483**	0.499**	0.503**
	p	0.001	0.001	0.001	0	0
*P< 0.05, **P < 0.001
